# Porcine adenovirus type 3 E1B^large ^protein downregulates the induction of IL-8

**DOI:** 10.1186/1743-422X-4-60

**Published:** 2007-06-12

**Authors:** Yan Zhou, Andrew Ficzycz, Suresh Kumar Tikoo

**Affiliations:** 1 Vaccine and Infectious Disease Organization, University of Saskatchewan, Saskatoon, Saskatchewan, Canada

## Abstract

Replication-defective (E1-E3 deleted) adenovirus vector based gene delivery results in the induction of cytokines including IL-8, which may contribute to the development of inflammatory immune responses. Like other adenoviruses, E1 + E3 deleted porcine adenovirus (PAdV) 3 induces the production of IL-8 in infected cells. In contrast, no IL-8 production could be detected in cells infected with wild-type or mutant PAdV-3s containing deletion in E1A + E3 (PAV211) or E1B^small ^+ E3 (PAV212). Expression of PAdV-3 E1B^large ^inhibited the NF-κB dependent transcription of luciferase from IL-8 promoter. Imunofluorescence and electrophoretic mobility shift assays suggested that constitutive expression of PAdV-3 E1B^large ^inhibited the nuclear translocation of NF-κB and its subsequent binding to DNA. These results suggest that E1B^large ^interacts with NF-κB to prevent transcription and down regulate proinflammatory cytokine IL-8 production.

## Background

Cytokines are important mediators of inflammation and regulators of the immune response. The inflammatory response including release of inflammatory cytokines is the first defense against viral infection. However, viruses have evolved a number of different strategies to avoid the host inflammatory responses. Large DNA viruses including poxviruses and herpes viruses [1-6] modulate cytokine action by encoding secreted forms of receptors for cytokines and chemokines. Adenoviruses modulate cytokine expression by encoding intracellular proteins, which counteract TNF-α [7,8].

Although human adenovirus (HAdV) vectors have been utilized for gene transfer for functional studies *in vivo *[9,10], their therapeutic use in delivering genes to the airways of humans is limited due to the transient gene expression [11]. Earlier studies have shown that the airway administration of adenovirus vector results in the induction of non specific host responses consisting in part of neutrophil accumulation followed by mononuclear cell and macrophage accumulation. Adenovirus vector infection of airway epithelial A549 cells [12,13] or airways of macaques [14] results in rapid induction of the inflammatory cytokine IL-8, which may contribute to the inflammatory host response [12]. This induction of IL-8 production has been shown to be due to adenovirus induced activation of Raf/MAPK pathway [15]. Thus, blocking these pathways may be required for developing an efficient adenovirus vector.

Porcine adenovirus (PAdV) 3, a non human adenovirus is being developed as a vector for gene delivery in animals and humans [16,17]. Availability of the complete nucleotide sequence and transcription map of PAdV-3 [18] genome has facilitated the construction of recombinant PAdV-3s [16,17,19,20] and their use as vaccine delivery vehicles [21]. Earlier, analysis of early region 1 (E1) of PAdV-3 suggested that while E1A [20] and E1B^large ^[19] are essential for virus replication, E1B^small ^is not essential for virus replication [20]. Here, we report that E1B^large ^can impair the induction of inflammatory cytokine IL-8 by inhibiting the NF-κB dependent gene transcription.

## Results and discussion

### RNase protection assay

**Figure 1:**
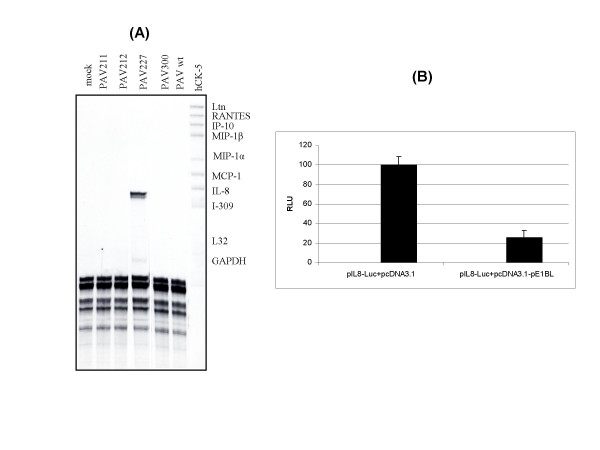
**PAdV-3 E1B^large ^inhibit IL-8 production. (A) **Total RNA isolated at 6 h post infection of HeLa cells with indicated viruses was analyzed by RNA protection assay using RiboQuant Multi-Probe template set hCK-5. The protected band indicated by the label on the right migrate faster that undigested probes, as expected.**(B).**HeLa cells transfected with the human IL-8 promoter containing a NF-κB recognition sequence, cloned upstream from a luciferase reporter cDNA in the presence of plasmid pCDNA3.1 or pCDNA3.1-pE1BL were assayed for luciferase activity (expressed as relative light units [RLU]). The error bars represent the standard error of mean of triplicate samples.

Earlier, induction of chemokines has been reported in adenovirus vector infected mouse renal epithelial cells [22], A549 cells [12] and HeLa cells [15], but not in U373 cells [7]. Moreover, both E1A and E3 gene products have been shown to down regulate the transcription of some chemokines [7,23]. To determine the effect of PAdV-3 E1 proteins on the induction of chemokines, HeLa cells were infected with PAV211 (E1A nt [530–1230] + E3 [nt 28112–28709] deleted), PAV212 (E1B ^small ^[nt 1460–1820] + E3 [nt 28112–28709] deleted), PAV227 (E1A + E1B^small ^+ E1B^large ^[nt 524–3274] + E3 [nt 28112–28709] deleted) or PAV300 (E3 [nt 28112–28709] deleted) at an MOI of 100 infectious units [24]. The construction and characterization of the mutant PAdV-3s has been described [19,20]. At 6 h post infection, the cells were harvested and processed for the isolation of total RNA using TRIZOL (Invitrogen) as per manufacturer's protocol. RNase protection assay was performed with the RiboQuant Muti-Probe template (BD Biosciences) set hCK-5 as per manufacturer's protocol. Autoradiographs were analyzed by a Molecular phosphoimager FX and Quantity One software (BIO-RAD). As seen in Fig. 1A, no chemokine specific transcript could be detected in the cells infected with wild-type or mutant PAdV-3 containing deletion of E3 (PAV300), E1A + E3 (PAV211) or E1B^small ^+ E3 (PAV212). Interestingly, IL-8 transcript was the dominant chemokine gene induced in the cells infected with recombinant PAdV-3 containing deletion of E1A + E1B^small ^+ E1B^large ^+ E3 (PAV227). These results suggest that E1B^large ^protein inhibit the expression of inflammatory cytokine IL-8.

### Luciferase reporter assay

Since increased expression of proinflammatory chemokines including IL-8, in response to various stimuli including adenovirus vectors can be upregulated by NF-κB transcription factor [22], we employed luciferase reporter assay to examine the inhibition of transcriptional activation of IL-8 promoter (containing consensus sequence for NF-κB binding) by E1B^large ^protein. As seen in Fig. 1B, reduced levels of the luciferase activity were obtained when phIL8-Luc DNA was cotransfected with pCDNA3.1-pE1BL DNA (expressing E1B^large^). In contrast, significant levels of luciferase activity were detected when phIL8-Luc DNA was cotransfected with pCDNA3.1 DNA showing that the competition for transcription factors binding to E1B^large^expression vector did not nonspecifically reduce the activity of luciferase reporter gene. The results of the reporter gene expression indicated that E1B^large ^reduced the NF-κB activated gene expression and was responsible for the observed inhibition of inflammatory cytokine IL-8 production.

### E1B^large ^inhibits the translocation of NF-κB to the nucleus

**Figure 2:**
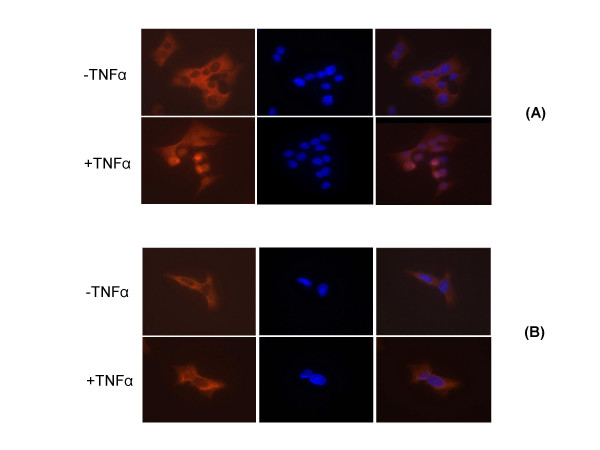
**Expression of NF-κB**. The cells constitutively expressing (VR1BL) or not expressing (VIDO R1) PAdV-3 E1B^large ^were treated with TNF-α. After 15 min, the cells were fixed with 100% methanol and analyzed by indirect immunostaining with anti- NF-κB p65 antibody followed by Cy™ conjugated goat anti-mouse secondary antibody. Finally, the cells were incubated with DAPI and visualized using Zeiss AxioVision microscope. **(A) **VIDO R1 cells, **(B) **VR1BL cells. DAPI (blue); NF-κB p65 (red).

NF-κB is a dimmer of two heterologous proteins (p65 and p50) held in an inactive complex by an endogenous inhibitor IκB, in the cytoplasm [25]. After cell activation, IκB is phosphorylated and subsequently degraded releasing NF-κB, which translocates to the nucleus where it binds to the enhancer elements upstream from the transcriptional initiation site of proinflammatory cytokine genes [25]. In order to determine if the expression of E1B^large ^alters the translocation of NF-κB to the nucleus, we analyzed the localization of p65 protein in VIDO R1 (fetal porcine retina cells expressing HAdV-5 E1A + E1B^small^)[17] or VR1BL (fetal porcine retina cells expressing HAdV-5 E1A + E1B^small ^and PAdV-3 E1B^large^)[19] cells using immunofluorescene assay. As seen in Fig. 2A, NF-κB is predominantly located in the cytoplasm of VIDO R1 cells [17]. As expected, TNF-α treatment translocated NF-κB to the nucleus of VIDO R1 cells. Similarly, NF-κB is predominantly located in the cytoplasm of VR1BL [19] cells (Fig 2B). However, TNF-α treatment did not alter the cytoplasmic location of NF-κB in VR1BL cells. These results suggest that the constitutive expression of PAdV-3 E1B^large ^is able to inhibit the translocation of NF-κB in TNF-α treated VR1BL cells.

### E1B^large ^affects the NF-κB binding to oligonucleotides containing NF-κB consensus sequence

**Figure 3:**
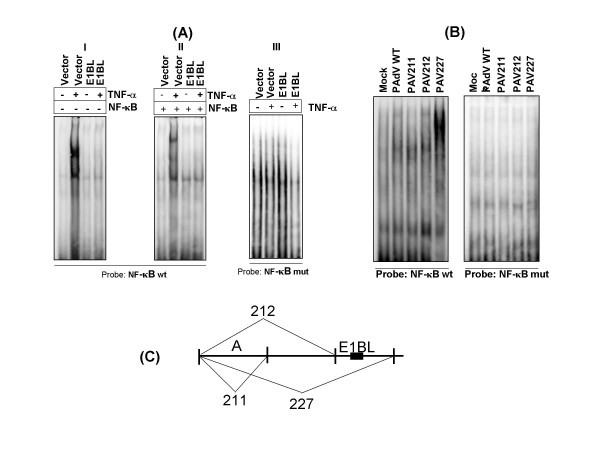
**EMSA of nuclear extracts. (A)**. Nuclear extracts from the plasmid transfected cells (I, II, III) incubated with radiolabeled oligonucleotide probe(s) containing wild-type (I, II) or mutant (III) NF-κB motif from human IL-8 promoter [30] with (II) or without (I) immunoprecipitation with anti- NF-κB p65 serum. **(B) **Nuclear extracts from mock infected or virus infected cells containing wild-type (I) or mutant (II) NF-κB motif from human IL-8 promoter. **(C) **Schematic diagram showing deletion of the regions in PAV211, PAV212 and PAV227 [19,20].

In order to investigate the effect of PAdV-3 E1B^large ^protein on binding of NF-κB protein to an oligonucleotide containing the IL-8 NF-κB DNA sequence [26,27], initially, we analyzed the nuclear extracts from transfected and nontransfected cells by electrophoretic mobility shift assay (EMSA). HeLa cells were transfected either with plasmid pCDNA3.1 DNA or with plasmid pcDNA3.1-pE1BL DNA as described above. At 48 h post transfection, the cells were left untreated or treated with TNF-α for 30 min before HeLa cell nuclear lysates were prepared as described previously [28]. The nuclear extracts were analysed by EMSA using labeled oligonucleotides containing wild-type NF-κB or mutant NF-κB. The results are shown in Fig. 3. As expected TNF-α treatment induced the binding of NF-κB to its consensus binding sequence in nuclear lysates of the cells transfected with plasmid pCDNA3.1 (panel A I). No such binding was observed following TNF-α treatment of the cells transfected with pCDNA3.1-pE1BL. Super shift assays using anti-NF-κB p65 antibodies demonstrated a supershifted band in the nuclear extracts of cells transfected with pCDNA3.1 DNA (panel A II). No such band could be observed when mutant NF-κB oligonucleotides were used as a probe with the nuclear extracts of the cells transfected with pCNDA3. or pCDNA3.1-pE1BL DNAs (panel A III). To further confirm these results, swine testicular (ST) cells were infected with wild-type or mutant PAdV-3s. At 6 h post infection, the infected cells were collected and the nuclear cell extracts prepared as described above. The nuclear extracts were analyzed by EMSA using wild-type or mutant NF-κB probe. As expected, NF-κB binding to oligonucleotides containing NF-κB consensus sequence could be detected in the nuclear extracts of the cells infected with PAV227 (Panel BI). No such binding could be detected when mutant NF-κB sequence was used with the nuclear extracts in EMSA (Panel BII). These results confirmed that E1B^large ^(panel C) mediated the inhibition of NF-κB translocation to the nucleus of the cell, hence preventing the NF-κB binding to NF-κB consensus sequences in the nucleus.

## Conclusion

**Figure 4:**
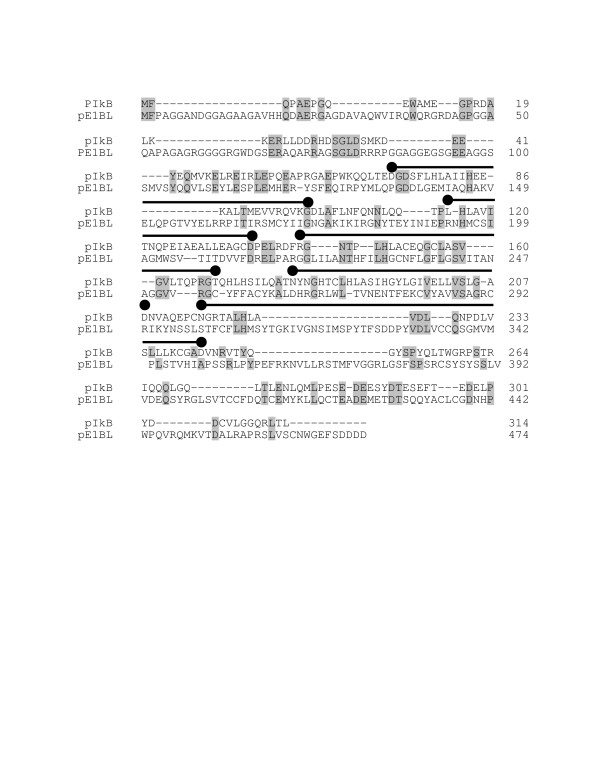
**Homology of E1B^large ^to IκBα**. Alignment of porcine IκBα (pIκB; GenBank Accession # A38490) and PAdV-3 E1B^large ^(pE1BL: GenBank Accession # AF083132). Shaded residues are identical between pIκB and pE1BL. Lines shown above denote the five repeats of ankyrin consensus sequence in IκBα.

In summary, we have demonstrated that PAdV-3 E1B^large ^protein downregulates the induction of proinflammatory cytokine IL-8 by inhibiting the NF-κB dependent gene transcription from human IL-8 promoter. Moreover, immunofluorescence and EMSA data suggest that the E1B^large ^protein inhibits the nuclear translocation of NF-κB by interacting with NF-κB. One possible mechanism of E1B^large ^action could be to act as IκB homolog and retain the ability to bind, and inactivate NF-κB. Interestingly, PAdV-3 E1B^large ^shows 20% identical and 38% homology (Fig. 4) at the amino acid level to porcine IκB protein (GenBank Accession # A38490). Similar homology is reported between African swine fever virus encoded IκB (A238L) protein and porcine IκB protein [29]. Alternatively, the nuclear localization of E1B^large ^[19] could have direct inhibitory effect on IL-8 transcription. These results suggest that the construction of adenovirus vectors to include E1B^large ^expression cassettes will improve the efficacy and safety of such vectors.

## Methods

### Viruses and cells

Recombinant PAdV-3 bearing deletions in the E1 region were generated as described previously [20]. PAV211 contains deletions in the E1A + E3 regions, PAV212 contains deletions in the E1B^small ^+ E3 regions, PAV227 contains deletions in E1 + E3 regions and PAV300 contains deletion in E3 region. Viruses were propagated and titrated as described [19,20,24]. HeLa cells were maintained in Dulbecco's modified Eagle's medium (DMEM) containing 10% fetal calf serum (FCS).

*RNase protection assays*. Monolayers of HeLa cells (1X10^5^/well) in 12 well plate were infected with wild-type or mutant PAdV-3s at a MOI of 100. At 6 h post infection, HeLa cells were harvested and processed for total RNA using TRIZOL (Invitrogen) as per manufacturer's protocol. RNase protection assay was performed with the RiboQuant Multi-Probe template (BD Biosciences) set hCK-5 as per manufacturer's protocol. Autoradiographs were analyzed by phosphoimaging with a Personal FX phosphoimager and Quantity One software (Bio-Rad).

### Plasmid construction

The 181-bp human IL-8 promoter sequence (-135 to +46) was PCR amplified from the genomic DNA [26,27] derived from HeLa cells using the primers: hIL8 (-135) Fw: 5'-CAAT**GCTAGC**G AAGTGTGATGACTCAGG TT-3', which contains a NheI restriction enzyme site (bold letters), and hIL8 (+46) Bw: 5'-CGTT**CTCGAG**A AGCTTGTGTGCTCTGCTGT-3' containing a XhoI restriction enzyme site (bold letters). The PCR product was digested with NheI-XhoI and ligated to NheI-XhoI digested plasmid pGL3-Basic (Promega) creating plasmid phIL8-Luc. The plasmid phIL8-Luc contains luciferase gene under the control of IL-8 promoter. Similarly, the coding region of E1B^large ^gene was PCR amplified using the primers: [PE1BL (NheI) Fw: 5'-CAG**TGCTAG**CATGTTCCCTGC TGGAGGCGC-3', which contains a NheI restriction enzyme site (bold letters), and PE1BL (XhoI) Bw: 5'-GTCA **CTCGAG**TC AGTCATC G TCATCGCTGAA-3' containing a XhoI restriction enzyme site (bold letters)] and PAdV-3 genomic DNA as a template. The PCR product was digested with NheI-XhoI and ligated to NheI-XhoI digested plasmid pCDNA3.1(-) (Invitrogen) creating plasmid pCDNA3.1-pE1BL. The plasmid pCDNA3.1-pE1BL contains E1B^large ^gene under the control of human cytomegalovirus immediate early (HCMV IE) promoter.

### Luciferase assay

HeLa cells (1x10^5 ^cells/well) were plated in 12-well plate and incubated overnight. Tansfections were carried out using 0.5 μg of each plasmid [(phIL8-Luc, pCDNA3.1-pE1BL] or [phIL8-Luc, pCDNA3.1])/well (in triplicate) using 5 μl of lipofectin (Invitrogen), followed by incubation for 5 h in Opti-MEM (Invitrogen). After adding FCS to each well to give a final concentration of 1%, the cells were incubated for 18 h at 37°C. Finally, the cells were washed with PBS and lysed in 200 μl of 1x lysis buffer (Luciferase reporter assay kit, BD Bioscience). Luciferase activity was determined using 50 μl of cell extract and was read using a TD-20/20 luminomitor (Turner Designs).

### Immunofluorescent Microscopy

VIDO R1 [17] and VR1BL [19] cells plated on glass coverslips were untreated or treated with 10 ng/ml TNF-α (R&D System). At 15 min post treatment, the cells were washed with PBS, fixed and permeabilized by incubating with methanol/acetone (1:1) at -20°C for 15 min. The cells were rehydrated with PBS and incubated for 1 hour in a 1:200 dilution of monoclonal antibody specific for the p65 subunit of NF-κB factor (Santa Cruz). The cells were washed three times with PBS and incubated with 1: 800 diluted Cy3-labeled goat anti-mouse antibody (Jackson Laboratory) for 30 min at room temperature. Finally, the cells were washed three times in PBS before incubating with DAPI at concentration of 1μg/ml (Roche) for 5 min. Fluorescence was examined and photographed using a Carl Zeiss Axiovert 200 M inverted fluorescent microscope.

### Electrophoretic mobility shift assays (EMSA)

HeLa nuclear lysates were prepared as described previously ([28]. Briefly, the cells were washed two times with phosphate-buffered saline, resuspended in 4 pellet volumes of buffer A [(10 mM TRIS (pH 7.9), 10 mM NaCl, 1.5 mM MgCl_2_, 5 mM dithiothreitol (DTT), 0.5 mM phenyl-methyl sulfanyl fluoride (PMSF), and 5 μg of aprotinin, leupeptin, and pepstatin (ALP) per ml)] and incubated at 4°C for 1 h. The cells were lysed by three freeze/thaw cycles and centrifuged for 5 min at 2000 × g at 4°C. The nuclei were washed once with buffer A, resuspended in 3 pellet volumes of buffer B [(20 mM TRIS (pH 7.9), 20% glycerol, 400 mM NaCl, 1.5 mM MgCl_2_, 0.2 mM EDTA, 5 mM DTT, 0.5 mM PMSF, and 5 μg of ALP per ml)] and incubated at 4°C for 30 min. The nuclear lysates were collected after centrifugation for 30 min at 12,000 × g at 4°C and stored at -80°C. The oligonucleotides containing wild-type NF-κB (shown in boldface) motif (5'-CGTAGCCATCAGTTGCAAA TCG**TGGAATTTCCT**CT-3') or mutant NF-κB (mutated residues underlined) motif (5'CTAGGCCATCAGTTGCAAATCG**TTTAATTTAAT**CT) [30] were end-labeled with [α-^32^P] dCTP using the Klenow fragment of DNA Polymerase I.

Each binding reaction was assembled on ice containing 0.2 ng of double-stranded labeled probe, 10 μg of HeLa nuclear lysate from indicated samples, 0.5 μg poly(dI-dC), 10 mM Tris (pH 7.8), 50 mM NaCl, 1 mM EDTA and 3.3 mM sodium acetate. DNA-protein complexes were electrophoresed for 2 h at 150 V through 5% acrylamide gels. The gels were dried for 60 min at 80°C and exposed to Phosphor screens. Images were analyzed with a Molecular phosphoimager FX and the Quantity One software package (BIO – RAD).

## Competing interests

The author(s) declare that they have no competing interests.

## Authors' contributions

YZ designed and carried out the experiments, and helped to analyze the data. AF designed, performed and helped to analyze the EMSA experiments. SKT helped to design the study and drafted the manuscript. All authors read, made corrections and approved the final manuscript.
